# Microzooplankton Growth Rates Examined across a Temperature Gradient in the Barents Sea

**DOI:** 10.1371/journal.pone.0086429

**Published:** 2014-01-24

**Authors:** Gayantonia Franzè, Peter J. Lavrentyev

**Affiliations:** Department of Biology, The University of Akron, Akron, Ohio, United States of America; Stazione Zoologica, Italy

## Abstract

Growth rates (µ) of abundant microzooplankton species were examined in field experiments conducted at ambient sea temperatures (−1.8–9.0°C) in the Barents Sea and adjacent waters (70–78.5°N). The maximum species-specific µ of ciliates and athecate dinoflagellates (0.33–1.67 d^−1^ and 0.52–1.14 d^−1^, respectively) occurred at temperatures below 5°C and exceeded the µ_max_ predicted by previously published, laboratory culture-derived equations. The opposite trend was found for thecate dinoflagellates, which grew faster in the warmer Atlantic Ocean water. Mixotrophic ciliates and dinoflagellates grew faster than their heterotrophic counterparts. At sub-zero temperatures, microzooplankton µ_max_ matched those predicted for phytoplankton by temperature-dependent growth equations. These results indicate that microzooplankton protists may be as adapted to extreme Arctic conditions as their algal prey.

## Introduction

A recent decline in sea ice cover over the Arctic, with the largest losses in the Eurasian sector, has resulted in areas of open water stretching from the shelves into the deep basins [1], [2]. Changes in the cryosphere can be gradual or abrupt [3], but they have cascading effects through polar ecosystems, including food web structure and elemental cycling pathways [4]. For example, 5°C might be a temperature threshold for Arctic marine ecosystems to become net heterotrophic [5]. Specific predictions about the trajectories of food web changes are complicated by the non-linear nature of their responses to climate change and, therefore, require a detailed knowledge of their key components and linkages to dynamic processes. These considerations warrant interest in the effects of climate change on microbial plankton because even minor effects at the base of food webs could be amplified through trophic chains [6].

Plankton growth rate is a fundamental biological property and governs species composition, productivity, and carbon transformations in pelagic systems [7]. Therefore, knowledge of growth rates of individual species and their assemblages is critical to understanding food web responses to climate change. Increasing sea temperatures will likely have different effects on growth rates of different functional and taxonomic groups within pelagic communities, including microzooplankton. The resulting compositional changes may, in turn, alter food web structure and trophic interactions [8]. For example, Rose and Caron [9] hypothesized that microzooplankton growth would be more constrained by low temperatures than phytoplankton growth. This hypothesis is based on growth-temperature curves extrapolated from laboratory cultures maintained at higher temperatures. However, field observations indicate that polar microzooplankton might be adapted to their extreme environment [10]. Landry and Calbet [11] suggested that mean instantaneous growth rates for microzooplankton in the ocean should be generally comparable to those of their phytoplankton prey based on biomass ratios. This assumption corresponds to earlier observations in temperate and tropical waters (e.g., [12]). However, the dearth of direct measurements of microzooplankton growth rates at low temperatures restricts our ability to extrapolate these estimates to polar systems.

In the Arctic, microzooplankton potential growth rates were examined at non-ambient temperatures in Disko Bay, Greenland [10], the Barents Sea [13], and a Spitsbergen fjord [14]. To date, the only experimental study of Arctic microzooplankton growth rates at sub-zero temperatures reported elevated rates for heterotrophic dinoflagellates as a group (up to 1.17 d^−1^; [15]). Clearly, more experimental data on microzooplankton growth and production rates in the Arctic are needed before we can predict and model their responses to climate change. Thus, the primary goal of the present study was to estimate growth potential of polar microzooplankton with a special emphasis on mixotrophic taxa. Specific objectives were to (1) determine growth rates of dominant microzooplankton species across a natural range of sea temperature variation in the Barents Sea; and (2) compare these rates to those based on published equations for ciliates and dinoflagellates. Although this study was not designed specifically to test the Rose and Caron hypothesis, we also compared the measured microzooplankton growth rates with phytoplankton growth rates predicted by several published temperature-growth equations.

## Materials and Methods

### Ethics Statement

Permission to sample in the Spitsbergen coastal waters was obtained from Svalbard authorities by the University of Tromsø, Norway (UiT). No specific permissions for other locations/activities were required. The field studies did not involve endangered or protected species.

### Study Locations

The Barents Sea is a large (1.4 million km^2^, average depth 230 m, maximum depth 500 m) polar shelf sea. It is the only Arctic region that remains unfrozen throughout the year up to 74–75°N [16], due to inflowing warm water masses of the Atlantic drift from the southwest [17]. The warm and more saline Atlantic water (AtW) subducts under the cold and fresher Arctic water (<0°C, ArW), which flows through the opening between Svalbard, Franz Josef Land, and Novaya Zemlia, and forms the distinct Polar Front between 74 and 76°N [18]. In addition to the Atlantic drift, the southwestern section of the shelf is affected by the coastal Nordcapp current.

**Figure 1:**
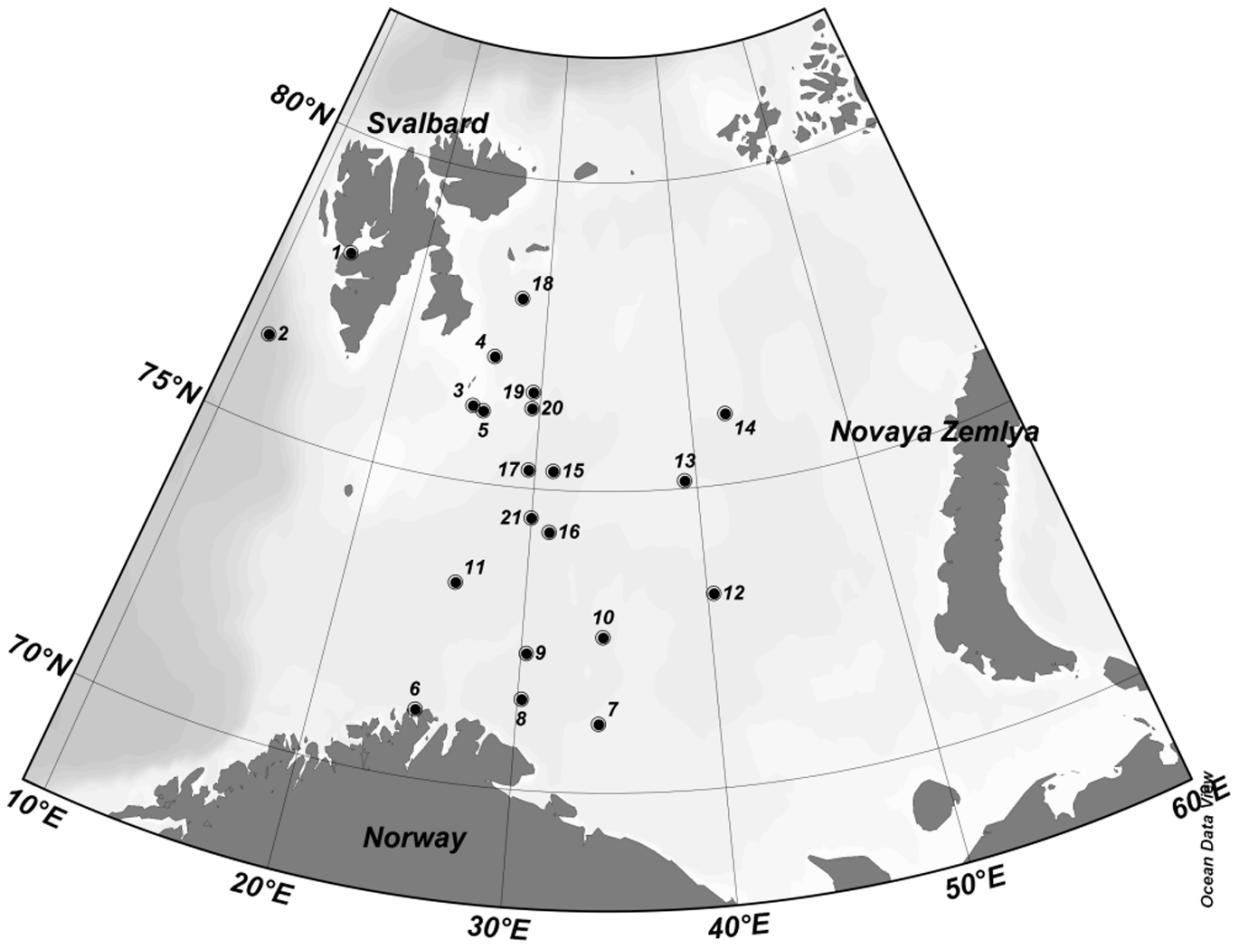
The study area with experimental station locations (corresponding to Table 1).

Twenty one field experiments were conducted at stations located between 70°N and 79°N and 11°E and 43°E in May 2010, August-September 2010, and June 2011 (Fig. 1) aboard the R/V Helmer Hanssen (formerly Jan Mayen) and R/V G.O. Sars. These expeditions, organized by the UiT and the Institute of Marine Research (Bergen, Norway), crossed the Polar Front from AtW in the south to ArW in the north. The UiT cruises (May 2010 and June 2011) also included the marginal ice zone and seasonal sea ice floes between Hopen Island and Kong Karls Land. In addition, experiments were conducted in Isfjorden, Spitsbergen, and the eastern slope of the Greenland Sea in May 2010.

### Field Sampling

Prior to sampling, all glass-ware, plastic containers, and tubing were soaked in 10% HCl and rinsed with copious amounts of deionized water and then seawater. Gloves were used whenever handling experimental containers. At each station, water temperature, salinity and raw fluorescence were measured using a Seabird 911 Plus CTD system equipped with a fluorometer. Seawater was collected in 5 L Niskin bottles from the fluorescence maximum depth (deep chlorophyll maximum, DCM) and carefully syphoned into a 20 L polycarbonate carboy using submerged silicone tubing. The carboy was immediately transported in an insulated cooler to a shipboard temperature-controlled cold room, which was adjusted to match (±1°C) the sea surface temperature. Additionally, sea ice cores were collected for Experiment (Exp.) 4.

### Experimental Manipulations

All manipulations were conducted under dim light, and samples were stored in a closed cooler whenever not being handled. The collected water was added carefully to triplicate 0.6 L Nalgene clear glass bottles. Additionally, in Exp. 4, the bottom 10 cm of sea ice was melted in unfiltered seawater at a 1∶8 ice to water (v/v) ratio to avoid osmotic shock mortality among protists [19], [20]. Growth experiments were run in conjunction with microzooplankton and copepod grazing experiments. Therefore, the samples were amended with dissolved nutrients to final concentrations of 16 µM N (KNO_3_+NH_4_Cl; 15∶1 based on N) and 1 µM P (K_2_HPO_4_). To avoid damaging delicate microzooplankton and altering phytoplankton composition, samples were not screened prior to incubation [15]. Instead, larger zooplankton such as copepods were removed using a glass pipette and headband flashlight. Post-incubation screening indicated that this technique was effective in removing mesozooplankton. The bottles were closed with Nalgene caps lined with Corning PTFE-faced silicone septa to prevent air headspace and screened with neutral density filters to mimic 25% surface irradiance.

### Sample Incubation

Bottles were incubated in a deck incubator with running surface seawater for 24 h. Temperature in the incubator was monitored manually using a handheld digital thermometer. During most experiments, temperature remained within ±0.5°C of the initial sea temperature (Table 1) since the ship was either at the station or moving within the same water mass. The only exception was Exp. 16 in September 2010, when the ship was called to port earlier than expected and crossed several temperature fields on the way back. Sea surface temperatures ranged from 4.6°C at the beginning of the incubation to 9.0°C at the end of this experiment. During the May 2010 cruise, the incubator included a plankton wheel, which was set at ca. 0.25 revolutions per minute [21]. During the other two cruises, experimental bottles were rotated periodically by hand to prevent phytoplankton settling.

**Table 1:** Experimental dates and conditions.

Exp	Date	Sea T°C	Incubator T°C	Sampling Depth m	Chl µg L^−1^
1	04/05/2010	−1.3	−1.2	17	1.64
2	05/05/2010	2.2	2.0	10	0.11
3	07/05/2010	−1.3	−1.3	10	3.19
4	08/05/2010	−1.8	−1.8	2	3.52
5	09/05/2010	0.3	0.1	35	1.37
6	24/08/2010	8.6	8.6	10	1.91
7	26/08/2010	7.2	7.2	10	1.82
8	27/08/2010	7.4	7.6	30	1.58
9	30/08/2010	7.5	7.5	20	1.20
10	01/09/2010	5.6	5.6	10	1.77
11	02/09/2010	7.0	7.0	25	0.79
12	04/09/2010	4.2	4.5	20	2.18
13	07/09/2010	3.1	3.1	20	0.67
14	09/09/2010	2.4	2.4	20	0.38
15	10/09/2010	4.9	4.9	20	0.10
16	12/09/2010	4.6	4.6–9	20	1.61
17	21/06/2011	4.0	5.0	35	1.86
18	22/06/2011	−1.8	−1.2	30	5.19
19	24/06/2011	−0.5	−0.8	44	1.32
20	26/06/2011	0.0	1.2	38	1.37
21	27/06/2011	4.0	4.8	20	3.00

**Legend:** T – temperature, Chl – chlorophyll a. Experiment sequential numbers correspond to Figure 1.

### Sample Collection and Preservation

Microzooplankton samples were collected from whole water treatments at the beginning and end of experiments, preserved in 2% (final concentration) acid Lugol’s iodine, stored at 4°C in 125 mL opaque containers, and post-fixed with 1% (final concentration) formaldehyde after 24 h. An additional set of triplicate plankton samples was fixed with 1% (final concentration) formaldehyde and stored as described above. For chlorophyll *a* (Chl) analysis, 250–500 mL of seawater was filtered onto 0.2 µm 47 mm nylon membrane filters, which were shock-frozen and stored in liquid N_2_. The samples were transported to the shore-based facility in insulated coolers with added cold packs.

### Sample Processing

In the laboratory, Chl was extracted in 90% acetone for 24 h at −20°C and measured using the non-acidic method [22] on a Turner Designs TD-700 fluorometer. Microzooplankton were settled onto Utermöhl chambers and counted under an Olympus IX-70 inverted microscope equipped with differential interference contrast (DIC), fluorescence, and a digital camera. The entire surface area of a chamber was scanned at 200×. Protists were identified tentatively to the lowest possible taxonomic level consulting Bérard-Therriault et al. [23], Kofoid and Campbell [24], Kofoid and Swezy [25], Matishov al. [26], Scott and Marchant [27], Steidinger and Tangen [28], and Strüder-Kypke et al. [29].

At least 40 individual cells within each abundant taxon were sized with an eyepiece micrometer at 400–600×. All ciliates were included in the counts, whereas dinoflagellates <15 µm in maximum dimension were not [30]. The smallest abundant ciliates in this study were ca. 15 µm, whereas dinoflagellates extended into the nanoplankton range. Microzooplankton biovolumes were calculated from their linear dimensions by approximating geometric shapes [31] and converted to carbon [32]. Tintinnid volumes were calculated based on their cell dimensions. Since iodine fixation masks photopigments, the formaldehyde-fixed samples were settled as described above, and ciliates and dinoflagellates were examined for the presence of chloroplasts using DIC and red autofluorescence of Chl (Olympus U-MSWG filter cube). This combination allowed simultaneous visualization of pigmented and non-pigmented cellular structures and allocation of microzooplankton into heterotrophs and mixotrophs (here pigmented ciliates and dinoflagellates). Recent literature indicates that all plastidic genera found in this study are capable of phagotrophy [33]–[36]. Some of the formalin-fixed cells were also post-stained with DAPI to visualize their nuclei for taxonomic purposes.

### Rate Calculations

Microzooplankton instantaneous population growth rates (µ, d^−1^) were determined from the initial (n_0_) and final (n_t_) abundances of each morpho-species and incubation time (*t*, d) assuming exponential growth (µ = ln (n_t_/n_0_)/t). Temperature dependency of protist growth was described using the Q_10_ coefficient (i.e., the factorial rate increase due to a temperature increase of 10°C, Q_10_ = (µ_1_/µ_2_)^10/(t2−t1)^, where µ_1_ and µ_2_ are growth rates determined at temperatures t_1_ and t_2_, respectively).

### Rate Comparison

The observed µ were compared to predicted maximum specific growth rates (µ_pred_) of ciliates and dinoflagellates using allometric equations available in the literature (Table 2, [9], [10], [12], [37], [38], [39], [41], [45], [46], [88], [89]). With the exception of two studies, which used natural plankton from the Kattegat [37] and Disko Bay, Greenland [10], these equations were based on the growth rates of cultured protists at 4 to 20°C and included their own temperature coefficients. Some of these equations yielded apparent “negative growth rates” (i.e., population decline) at sub-zero temperatures [38], [39]. Therefore, the rates calculated using these equations for 10°C were converted to ambient temperatures using a Q_10_ of 2.8 [40]. The same Q_10_ coefficient was applied to equations for Disko Bay ciliates and dinoflagellates grown at 1.4°C [10]. The dinoflagellate and phytoplankton size-dependent growth equations were also converted to ambient temperatures using a Q_10_ of 1.58 [41]. In addition, the observed µ of microzooplankton were log_2_-transformed and compared with the temperature-dependent µ_pred_ of herbivorous microzooplankton [9] and phytoplankton [42]–[44]. The Bissinger et al. equation [42] is based on a larger data set (n = 1,501 vs. 162 in Eppley [43]), relies on quantile regression analysis vs. fitting the upper envelope by eye, and includes some data obtained at low temperatures. Nevertheless, all three phytoplankton growth-temperature equations share the same slope and differ only by their intercepts. The original Eppley [43] equation expressed growth in doublings d^−1^. Therefore, growth rates calculated with the latter formula were converted to µ (d^−1^) by multiplying doublings by log_2_.

**Table 2:** Published relationships between plankton growth rates (µ, d^−1^), temperature (T, °C), and cell volume (V, µm^3^) unless noted otherwise.

Equation	Source	Remarks
**Ciliates**		
Log_2_ µ = (1.52 Log_2_T) −0.27 Log_2_V −1.44	Muller and Geller [38]	
Log_2_ µ = 0.1438T −0.3285 Log_2_V −1.3815	Montagnes et al. [88]	V = µm^3^ 10^−3^
Log_2_ µ = 0.85 Log_2_ T −0.08 Log_2_V −1.34	Perez et al. [39]	
µ = 3.18 V^−0.243^exp (0.095T)	Nielsen and Kiørboe [37], [45]	
µ = 0.1248 V ^−0.331^	Levinsen et al. [10]	µ (h^−1^)
**Dinoflagellates**		
Log_10_ µ = −0.51295−0.243631 Log_10_V	Hansen [89]	µ (h^−1^)
µ = 0.0479 V^−0.25^	Nielsen and Kiørboe [37]	µ (h^−1^)
	after Levinsen and Nielsen [46]	
µ = 2.26C^−0.18^	Tang [41]	C = pg C
Log_10_ µ = 0.14−0.15 Log_10_C	Banse [12]	C = pg C
**Herbivorous Protists**		
Log_2_ µ = 0.10T −1.0	Rose and Caron [9]	
**Phytoplankton**		
Log_10_ µ = 0.0275T −0.07	Eppley [43]	(doublings d^−1^)
µ = 0.81 e^0.0631T^	Bissinger et al. [42]	
µ = 0.97 e^0.0633T^	Brush et al. [44]	
µ = 3.45C^−0.21^	Tang [41]	C = pg C

### Statistical Analyses

Rare taxa (here, less than 20 cells L^−1^ in the initial sample) were excluded from calculations to avoid statistically unreliable rate estimates. In several cases we settled additional Lugol-fixed samples, which were originally collected for phytoplankton analysis. Only those experiments where effect size was large enough (Cohen’s D >0.5) are reported. Effect size was estimated using SPSS. Standard error (± SE) is used as a measure of dispersion throughout the text. Experimental and field data were analyzed via quartile box plots, Student’s t-test, analysis of variance (ANOVA), Tukey’s multiple comparison of means, Pearson product-moment correlations, and linear regression using Minitab 16.

## Results

### Environmental Data

Sea surface temperatures ranged from −1.8°C in ArW under ice in May and June to 9.0°C in the Nordcapp current-influenced AtW off the coast of Finnmark in August (Table 1) and were inversely related to latitude (r = −0.91, p<0.001). Three shelf regions were distinguished based on sea temperature: ArW (below 0°C, average −1.30±0.24°C ), AtW (0–5°C, 3.41±0.74°C), and AtW>5 (above 5°C, 7.22±0.40°C).

**Figure 2:**
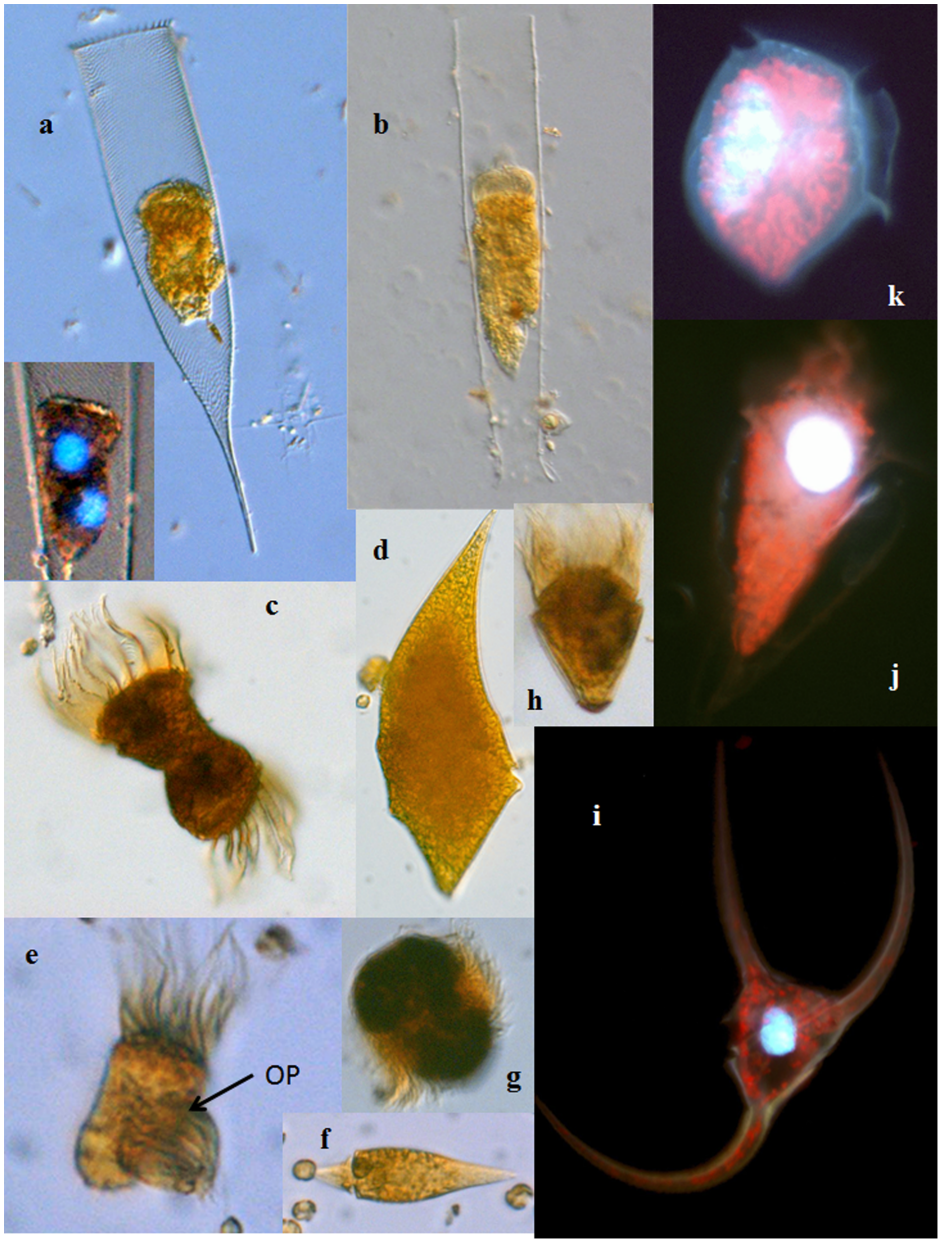
Common microzooplankton from the Barents Sea: (a) *Parafavella gigantea*, 250 µm (insert shows a mitotic cell stained with DAPI); (b) *Leprotintinnus pellucidus*, 300 µm; (c) cytokinetic *Strombidium* sp. 40 µm; (d) *Gyrodinium spirale* 120 µm*;* (e) cytokinetic *Lohmaniella oviformis* 22 µm, OP = oral primordium; (f) *Amphidinium sphenoides* 35 µm; (g) *Mesodinium rubrum* 35 µm; (h) *Strombidium constrictum* 40 µm; (i) *Ceratium arcticum* 200 µm; (j) *Strombidium conicum* 75 µm; (k) *Dinophysis norvegica* 65 µm. Images are not to scale. (a–h) Lugol’s-fixed cells under DIC; (i–k) DAPI and Chl autofluorescence.

Chl ranged from 0.10 µg L^−1^ on the Atlantic side of the Polar Front in September 2010 to 5.19 µg L^−1^ under the ice in June 2011. The average Chl during the study period was 1.74±0.27 µg l^−1^ (3.07±0.72, 1.40±0.31, 1.51±0.18 in ArW, AtW, and AtW>5, respectively). In Exp. 4, the ice cores contained chains of the diatom *Nitzschia frigida*. Under the ice, phytoplankton communities were dominated by large, chain-forming diatom species (*Thalassiosira* spp, *Fragilariopsis cylindrus, Rhizosolenia styliformis*), whereas most open water communities consisted primarily of nanoplankton-sized diatoms and nano- and picoflagellates. In June 2011, the colonial prymnesiophyte *Phaeocystis pouchetii* was also abundant in some samples collected in the marginal ice zone. Ciliate and dinoflagellate (>15 µm, Fig. 2) abundances ranged from 0.41 to 13.6 cells 10^3^ L^−1^ and 0.44 to 7.26 cells 10^3^ L^−1^, respectively.

### Species-Specific Growth Rates

Growth rates were measured for 14 species of aloricate ciliates, 2 species of tintinnids, 7 species of athecate dinoflagellates, and 8 species of thecate dinoflagellates (n = 163, Table 3). No single species grew in all experiments, but several taxa exhibited growth across nearly the entire temperature gradient (*Amphidinium sphenoides*, *Balanion comatum*, *Gymnodinium arcticum, G. simplex, Gyrodinium pingue, Gyrodinium spirale, Leegaardiella sol, Mesodinium pulex, M.rubrum, Strombidium conicum, S. epidemum, S. lynii*). The fastest species-specific growth rates (µ_max_) of microzooplankton in this study were measured at 4.5°C in September 2010 (Exp. 12), although several ciliates achieved their highest growth in ArW under the ice at −1.3°C. In two experiments (5 and 9), ciliates failed to grow. Overall, µ_max_ of ciliates ranged from 0.33 d^−1^ for *Lohmaniella oviformis* to 1.67 d^−1^ for *M. rubrum*. The µ_max_ of dinoflagellates varied between 0.52 d^−1^ (*Gymnodinium heterostriatim*) and 1.14 d^−1^(*G. simplex*).

**Table 3:** Growth rates and volumes of common microzooplankton species in the Barents Sea.

	Length	Volume (µm^3^ 10^−3^)	Growth rate (µ, d^−1^)		T (°C) max µ	n
Species	(µm)		max	min		
**Ciliates**						
*Uronema* sp.	15.0	0.79	0.68	0.21	−1.8	3
*Cyclotrichium sphaericum*	55.0	87.1	0.84	0.35	−1.3	3
*Parafavella gigantea*	70.0	74.2	0.46	0.40	−1.3	2
*Strombidium cf. coronatum*	65.0	18.5	0.74	0.33	−1.3	2
*Leegaardiella sol*	33.0	18.8	0.89	0.29	−0.5	4
*Strombidium epidemum*	20.0	2.36	0.97	0.14	−0.5	3
*Lohmaniella oviformis*	21.5	3.80	0.33	0.10	1.2	5
*Strombidium* sp.	22.0	2.95	0.80	0.54	2.0	3
*Parafavella obtusangula*	60.0	28.3	0.96	–	4.5	1
*Laboea strobila*	120	157	0.71	0.19	4.5	3
*Mesodinium pulex*	15.0	1.77	0.83	0.08	4.5	9
*Mesodinium rubrum*	35.0	16.5	1.67	0.28	4.5	10
*Strombidium cf. lynni*	30.0	8.17	1.06	0.07	4.5	7
*Strombidium wulfii*	47.0	15.3	1.31	0.53	4.5	5
*Balanion comatum*	20.0	2.35	0.72	0.20	6.8	7
*Strombidium conicum*		26.3	0.83	0.51	6.8	4
**Dinoflagellates**						
*Gymnodinium arcticum*	22.0	2.59	0.66	0.13	−1.3	14
*Gyrodinium spirale*	100	83.8	0.76	0.11	−1.3	7
*Gymnodinium heterostriatum*	75.0	91.6	0.52	0.23	0.1	4
*Dinophysis norvegica*	70.0	74.2	0.72	0.12	4.5	5
*Dinophysis rotundata*	40.0	18.8	0.81	0.11	4.5	3
*Gymnodinium simplex*	15.0	0.63	1.14	0.40	4.5	4
*Protoperidinium bipes*	28.0	8.18	0.90	0.13	4.5	6
*Torodinium* sp.	25.0	2.95	0.69	0.18	5.0	3
*Amphidinium sphenoides*	30.0	3.53	0.84	0.06	6.8	9
*Gymnodinium* sp.	40.0	18.8	0.76	0.12	6.8	11
*Gyrodinium pellucidum*	25.0	6.29	0.84	0.34	7.0	3
*Ceratium arcticum*	200	113	0.75	0.39	7.2	6
*Protoperidinium depressum*	100	335	0.63	0.19	7.5	6
*Gyrodinium pingue*	40.0	8.38	0.59	0.16	7.6	6
*Scripsiella trochoidea*	20.0	4.23	0.80	0.33	8.6	5

**LEGEND:** n = number of incubations, where the rates were measured.

**Figure 3:**
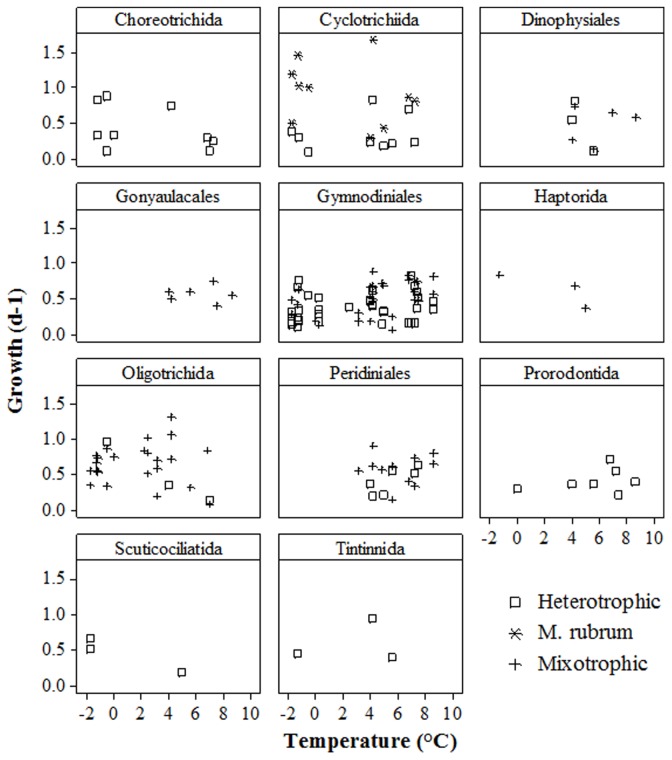
Microzooplankton growth rates within taxonomic orders across a temperature gradient.

The average Q_10_ for microzooplankton µ_max_ at −1.3°C and 4.5°C (Table 3) was 1.64 (1.79 for ciliates and 1.48 for dinoflagellates). For *M. rubrum*, Q_10_ was 1.36 within the same temperature range. The µ values did not correlate with temperature (p = 0.5) or cell volume (p = 0.5 and 0.17, respectively) in either phylum. However, all ciliates except *Strombidium conicum* reached their µ_max_ at temperatures <5°C, whereas half of the measured µ_max_ for dinoflagellates occurred in AtW>5. This trend also held for the entire data set when microzooplankton were arranged into taxonomic orders (Fig. 3). The thecate dinoflagellates from the orders Dinophysiales, Gonyaulacales, and Peridiniales grew only in AtW>5, whereas most ciliates grew more slowly at these temperatures than in the ice-covered ArW waters. Athecate dinoflagellates in the order Gymnodiniales grew in most experiments. Neither heterotrophic nor mixotrophic species from this order displayed temperature growth dependency.

**Figure 4:**
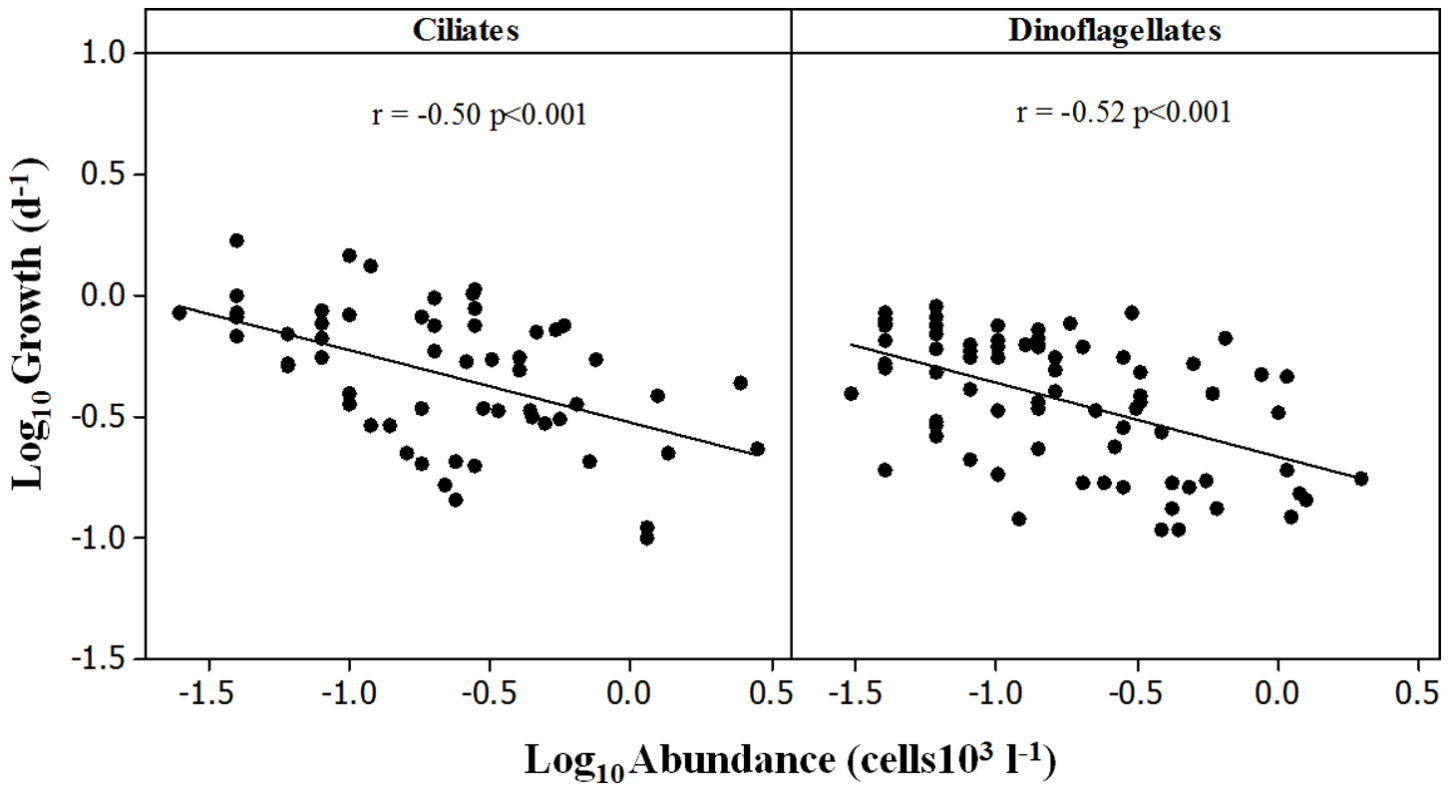
The relationship between the initial abundance and growth rates of microzooplankton species in bottle experiments.

The average µ of mixotrophic ciliates (excluding the fast growing *M. rubrum*) exceeded those of their heterotrophic counterparts in both ArW (0.62±0.05 d^−1^ vs. 0.47±0.07 d^−1^) and AtW (0.66±0.08 d^−1^ vs. 0.40±0.05 d^−1^). The same trend was found for dinoflagellates in AtW (0.55±0.03 d^−1^ vs. 0.41±0.05 d^−1^), whereas their average growth rates did not differ (0.34±0.08 d^−1^ vs. 0.33±0.05 d^−1^) in ArW. In both ciliates and dinoflagellates, species-specific growth rates were inversely related to the initial abundance (Fig. 4).

### Comparison with Predicted Size- and Temperature-dependent Growth Rate

**Figure 5:**
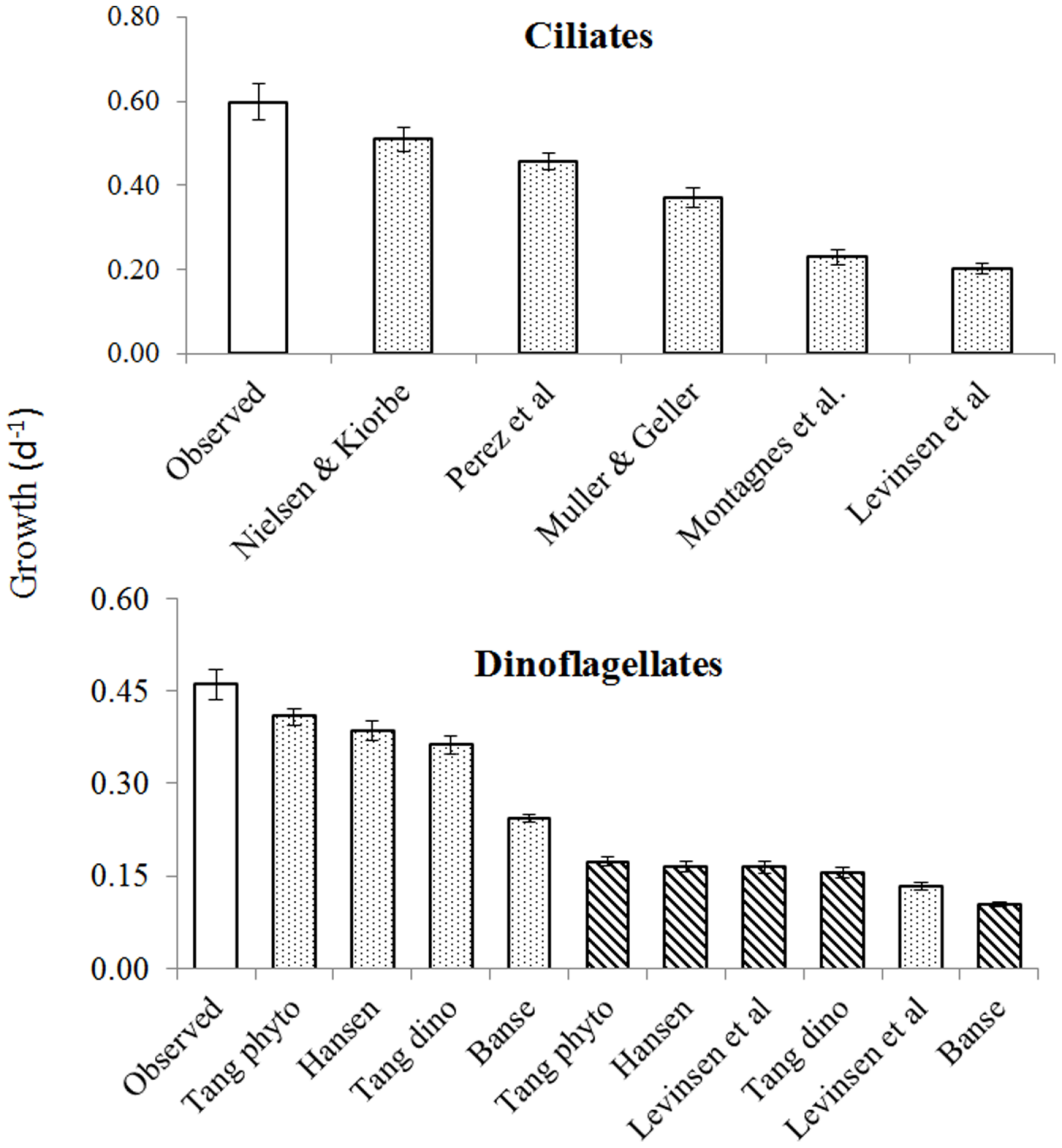
Comparison of the average observed and predicted growth rates. The equations used for growth rate calculations are presented in Table 2. Open bars indicate observed rates, striped bars indicate calculated rates corrected using Q_10_ = 2.8, dotted bars show Q_10_ = 1.58.

The average µ_pred_ estimated according to Nielsen and Kiørboe [37], [45] was the only one that overlapped with the average observed µ for ciliates (Fig. 5; Tukey’s test). However, these authors specifically excluded small ciliates from their equation. Therefore, the µ for species under 20 µm were compared with the next nearest µ_pred_ based on the Pérez et al. [39] equation in subsequent analyses. The Tang [41] equation for phytoplankton adjusted for ambient temperature using a Q_10_ of 1.58 was the closest fit for the observed µ of dinoflagellates, whereas all tested dinoflagellate formulae underestimated the observed µ (p<0.001) within the given cell size and sea temperature range.

**Figure 6:**
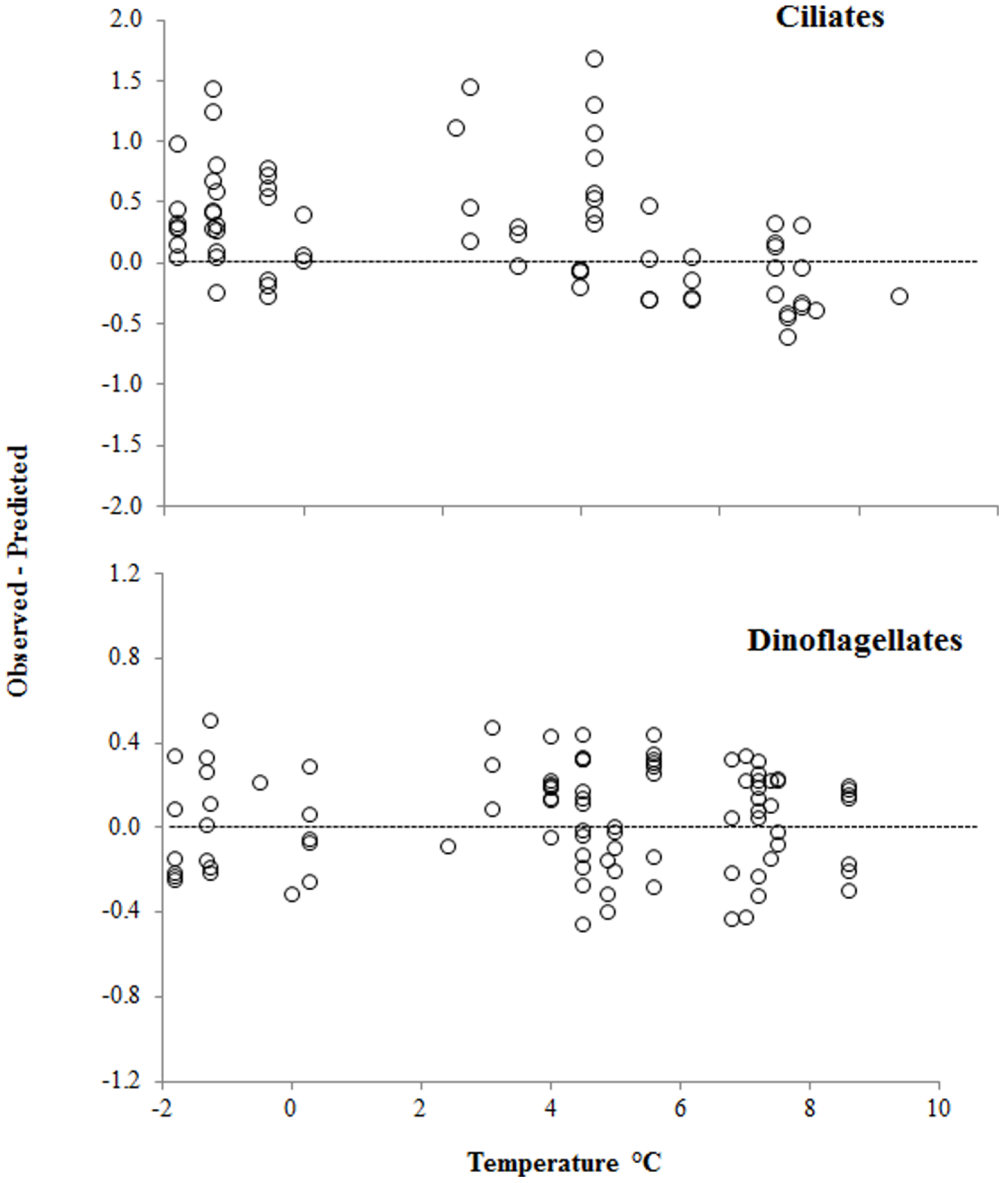
Differences between observed species-specific and predicted growth rates of ciliates and dinoflagellates across a temperature gradient. Predicted rates were calculated based on equations from Nielsen and Kiørboe [37], [45], Perez et al. [39], and Tang [41] for ciliates >20 µm, ciliates <20 µm, and phytoplankton, respectively.

**Figure 7:**
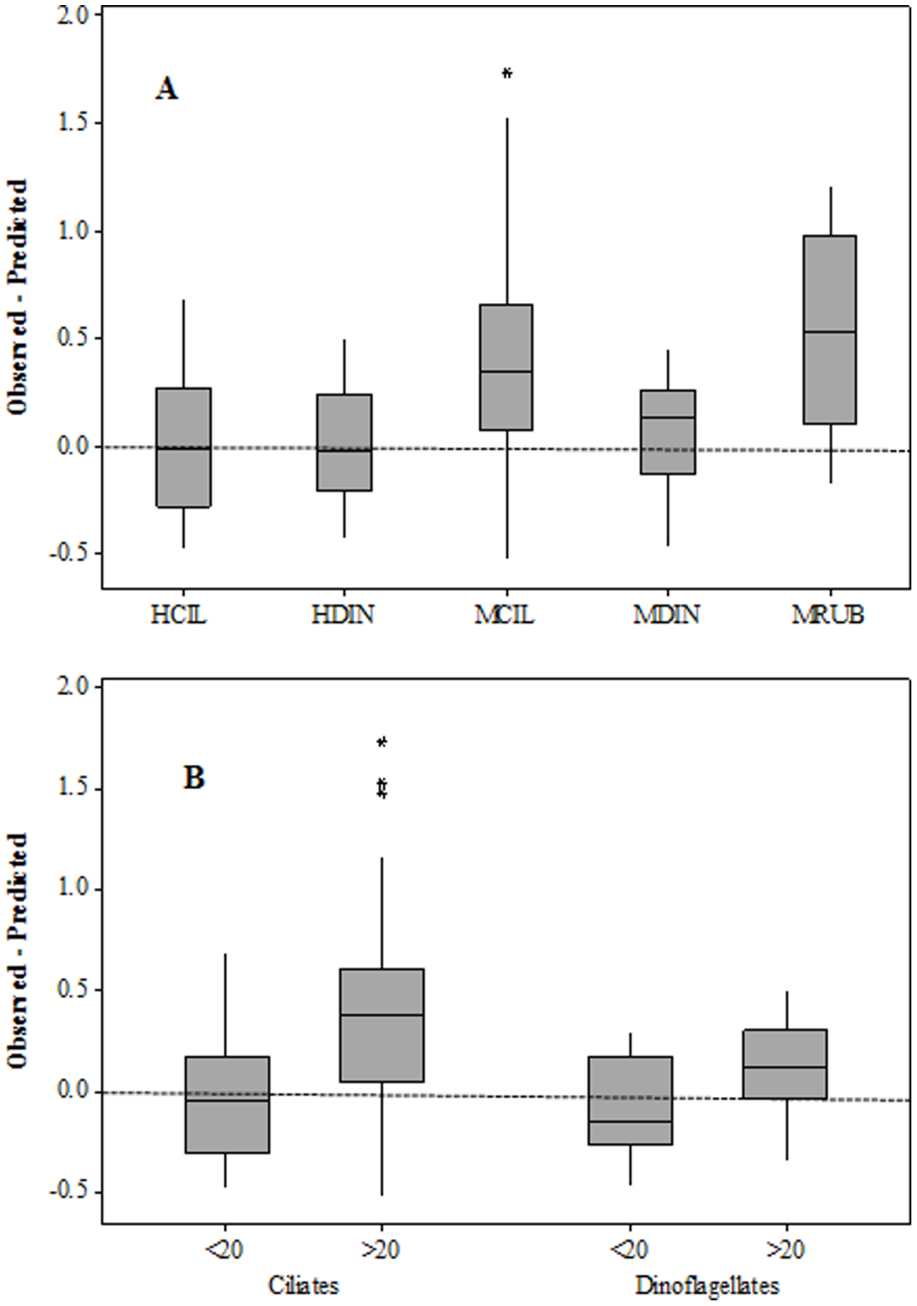
Average differences between observed species-specific and predicted growth rates from Figure 5 separated into microzooplankton functional-taxonomic groups (A) and size-taxonomic groups (B). HCIL = heterotrophic ciliates, HDIN = heterotrophic dinoflagellates, MCIL = mixotrophic ciliates, MDIN = mixotrophic ciliates, MRUB = *Mesodinium rubrum*.

Based on the above two formulae for ciliates, the observed species-specific µ were equal to or exceeded µ_pred_ at temperatures <5°C and lower than predicted in AtW>5 (Fig. 6a). This trend was not apparent in dinoflagellates, which exceeded µ_pred_ across the entire temperature range (Fig. 6b). Regardless of temperature, the median values of heterotrophic taxa µ were equal to their µ_pred_, whereas mixotrophic taxa, especially ciliates, grew faster than predicted (Fig. 7a). Likewise, large cells from both phyla grew faster than predicted, whereas the smaller ones did not (Fig. 7b).

**Figure 8:**
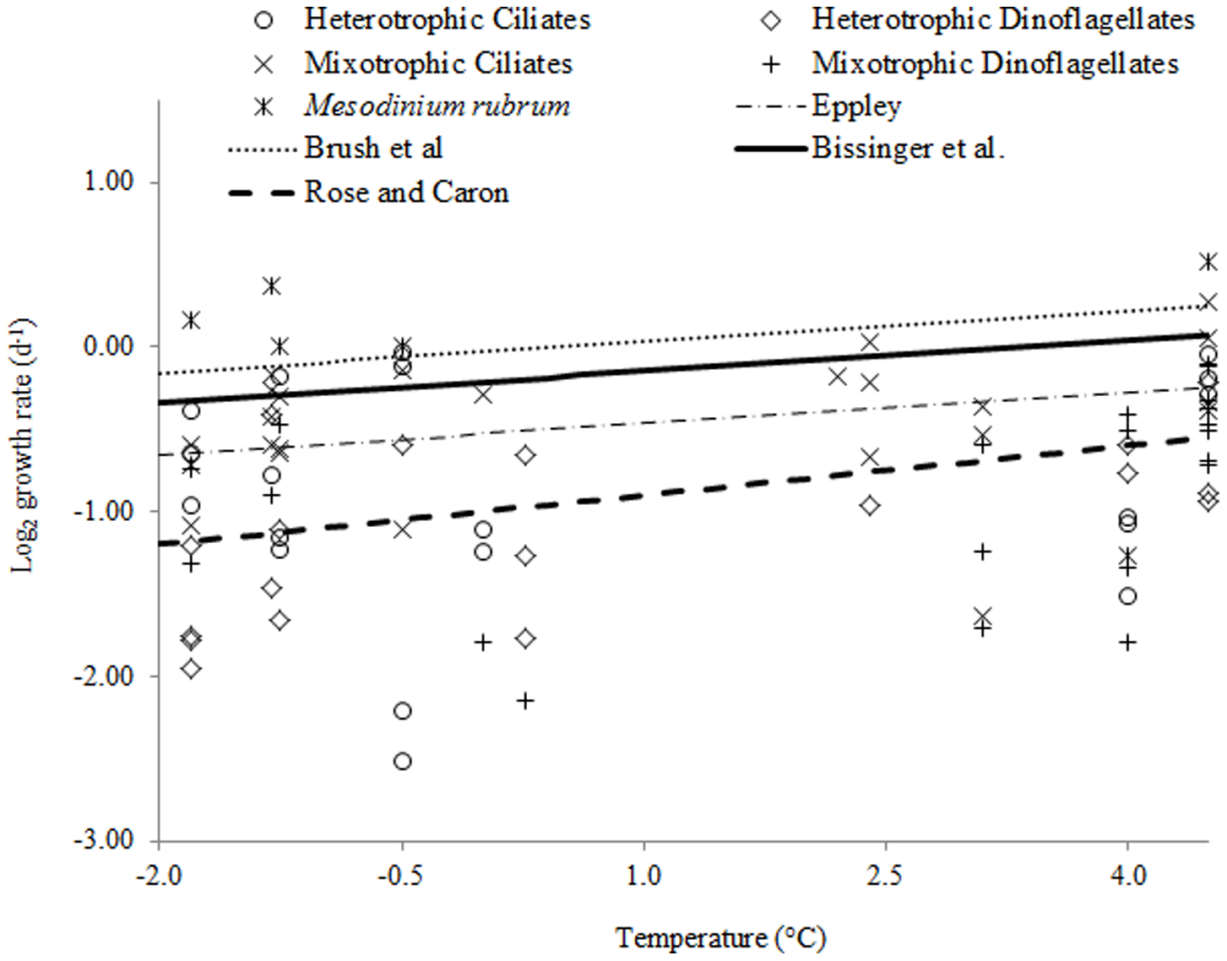
Observed species-specific growth rates of microzooplankton at temperatures below 5°C and predicted temperature-dependent growth rates of phytoplankton and herbivorous microzooplankton based on published equations from Table 2.

Microzooplankton grew at rates that were close or equal to the highest phytoplankton µ_max_ estimates between −1.8 and 4.5°C (Fig. 8). At temperatures <0°C, nearly 50% and 78% of the observed growth rates of microzooplankton exceeded the values predicted by Eppley [43] and Rose and Caron [9], respectively. With increasing temperatures, these ratios decreased to 21% and 52% between 0 and 5°C, and 0% and 22% at >5°C, respectively. The only species that exceeded the growth rates predicted by Brush et al. [44] was *M. rubrum*.

## Discussion

Few attempts have been made to measure microzooplankton growth rates in the Arctic due to logistical and methodological constraints. To our knowledge, this is the first field study to experimentally examine species-specific growth of polar microzooplankton across a broad natural temperature gradient. Maximum *in situ* growth rates of ciliates and dinoflagellates observed at sub-zero sea temperatures confirm the hypothesis that they are well adapted to their cold environment [10]. At the same time, many microzooplankton species from the Barents Sea appear to be eurythermal within the experimental temperature range. Although thecate dinoflagellate growth was restricted primarily to AtW>5, most ciliates and gymnodiniid dinoflagellates had wide temperature tolerances for growth. Eurythermy was also suggested for polar autotrophic protists [47]. It remains to be explored, however, whether the microzooplankton morphospecies described in this study are genetically divergent in the Arctic and Atlantic waters. For example, distinct polar clades were found within chlorophytes [48], [49].

For the experiment where incubation temperature increased from 4.6°C to 9°C in less than 24 h (Exp. 16), microzooplankton neither declined precipitously nor grew significantly faster than in most other experiments. This observation supports the potential rates obtained under altered conditions in previous Arctic studies. For example, ciliates from the −1.8°C ice-covered Arctic waters in the Barents Sea were incubated at 5±2°C without prior acclimation and grew at 0.47 to 1.38 d^−1^ [13]. Likewise, ciliates and dinoflagellates grew at rates up to 0.54 d^−1^ and 0.72 d^−1^, respectively, in samples collected from 0.5°C water in a Svalbard fjord and incubated at 2°C in the dark [14]. In Disko Bay, Greenland, ciliates and dinoflagellates collected from 3–7°C seawater were incubated at 1.4°C and achieved µ up to 0.3 d^−1^ and 0.49 d^−1^, respectively [10]. Combined, these observations indicate that Arctic microzooplankton can be resilient to abrupt and significant disturbances to their physical environment.

### Observed vs. Predicted Growth

Nearly 60% of the measured growth rates of microzooplankton in this study (70% at temperatures below 5°C) exceeded predictions based on temperature-extrapolated data from laboratory cultures. The main source of discrepancy between the observed and predicted µ_max_ appears to be the lack of culture data at the low temperature end. It is revealing, however, that a field-derived equation [37] provided a closer match to the observed rates of ciliates than those based on laboratory cultures. Thus, low temperature adaptations in Arctic microzooplankton may offer only a partial explanation for the observed vs. predicted rate mismatch, since all ciliate equations used in this study shared essentially the same Q_10_. Culture studies offer indisputable advantages, such as the ability to control growth conditions and isolate specific factors. However, they may select for clones that are acclimated to grow on specific food sources under laboratory conditions, which may not be optimum. For example, the highest growth rate recorded for *M. rubrum* in culture was 0.52 d^−1^ [35], whereas the µ_max_ of its polar strain did not exceed 0.2 d^−1^ [50]. However, a wild population of *M. rubrum* achieved a µ_max_ of 4.2 d^−1^ during the initial stages of a “red water” event in the Columbia River estuary [51]. Further, steady food supply and stable conditions in culture may not necessarily elicit a maximum growth response in microzooplankton. Under natural conditions, protists are adapted to survive on fluctuating and spatially heterogeneous resources [52], [53]. As a result, selection may favor those clones that can achieve their intrinsic maxima rapidly, when growth conditions improve. Therefore, *in situ* experiments might yield different and, probably, more reliable inferences of underlying temperature relationships for natural populations than data derived from laboratory experiments with individual cultures [8].

### Mixotrophic Growth

Rapid growth rates of mixotrophs in this study contrast with data from the Mediterranean Sea, where mixotrophic oligotrichs grew more slowly than their heterotrophic counterparts [39]. Mixotrophy can be a response to oligotrophic conditions, especially in larger cells, because photosynthetic carbon could cover a significant fraction of their metabolism due to lower volume-specific respiration rates [54]. However, large mixotrophic oligotrichs, such as *Laboea strobila*, dominated under phytoplankton bloom conditions in the Bering Sea [55]. Given the same prey concentration, a mixotrophic oligotrich grew faster under luxury light [56], whereas the proportion of phagotrophic and autotrophic-derived carbon in a mixotrophic dinoflagellate diet changed dynamically in response to light conditions and food availability [57].

In some experiments in this study, mixotrophic microzooplankton could have taken full advantage of the abundant prey and 24 h insolation. Samples collected from Chl maxima were exposed to irradiance levels that were approximately double their ambient levels, assuming a light attenuation coefficient of 0.09 m^−1^ [58]. Improvements in the light environment were even stronger for samples collected from under the ice. It is likely, however, that mixotrophic species encounter ambient growth conditions similar to those simulated in our experiments. Mixotroph abundance often peaked in the upper part of the mixed layer in this and previous studies in the Barents Sea [59], [60], and the DCM was within the range of their diel vertical migration [61], [62].

### Microzooplankton vs. Phytoplankton Growth

Our data do not support the Rose and Caron contention [9] that microzooplankton growth is more limited by low temperature than that of phytoplankton. This lack of congruence does not necessarily disprove their hypothesis but suggests that it should be approached with caution until more *in situ* rate data are collected at polar temperatures for both herbivores and phytoplankton. In fact, in another set of experiments these authors reported that ingestion rates of Antarctic ciliates were not constrained by low temperatures [63]. It should be noted that the temperature-dependent growth equation for herbivorous protists in the original Rose and Caron study included only cultured heterotrophs grown at temperatures >4°C, whereas Arctic microzooplankton endure much lower sea temperatures and usually include a large mixotrophic component [59], [64]–[67]. Nevertheless, the observed µ_max_ of heterotrophic ciliates and dinoflagellates in the present study were equal to predicted phytoplankton µ_max_ at temperatures below 4°C. Further, the average species-specific growth rate of microzooplankton below 0°C was nearly identical to the average µ_max_ of diatom isolates from the Barents Sea grown at −0.5°C (0.50±0.02 d^−1^, [68]).

Simultaneous measurements of microzooplankton and phytoplankton growth rates at ambient temperatures in the Arctic are scarce, yet they provide support for the above conclusion. Microzooplankton and phytoplankton average net growth rates based on one year of weekly biomass records in Disko Bay were comparable [64]. These authors noted that microzooplankton responded almost instantly to the spring diatom bloom and increased 100 fold from their winter minimum, despite sub-zero temperatures and predation by *Calanus*. In the Fram Strait, the heterotrophic dinoflagellate growth rate of 1.17 d^−1^ at −1.2°C [15] also exceeds predicted phytoplankton µ_max_ at this temperature based on Bissinger et al. [42]. Finally, microzooplankton growth rates calculated from their herbivory rates in dilution experiments exceeded those of phytoplankton in the Bering Sea at −1.6–4°C [69]. Although the latter estimates depend on a C:Chl ratio assumption and did not include mixotrophic dinoflagellates, it is likely that they were at least equal to phytoplankton growth. It should be noted that Arctic phytoplankton also may grow faster than predicted based on temperature. For example, diatoms grew as fast as 0.83 d^−1^ and 1.49 d^−1^ at 0°C and 5°C, respectively, in the Greenland Sea [15].

The slope of the Bissinger et al. equation yields a Q_10_ of 1.88, which is somewhat higher than Q_10_ coefficients based on phytoplankton growth in cultures (1.58 for 5–25°C, Tang 1995) and microzooplankton growth in this study (1.63 for −1.3–4.5°C). The latter value is also much lower than a Q_10_ of 2.6 estimated by Nielsen and Kiørboe for ciliate growth rates observed between 5 and 20°C in temperate waters [37]. Differences between these coefficients likely result from different temperature intervals in our and previous studies and are inherent in the Q_10_ approach. However, these differences may be meaningless if growth responses to changing temperature are linear rather than exponential as suggested by Montanges et al. [70]. These authors criticized the application of two-point Q_10_ to growth estimates for introducing a systematic error and neglecting the underlying complexities of the process. Instead, they proposed a solution where planktonic protists, including ciliates and diatoms, respond to temperature linearly with a single slope (0.07 d^−1^).

If we scale the average µ_max_ of herbivorous ciliates grown at 20°C (2.50±0.07 d^−1^, [9]) to −0.5°C using the above slope, the resulting rate of 1.06 d^−1^ will be similar to the observed rate (0.97 d^−1^) for the heterotrophic oligotrich ciliate *Strombidium epidemum* in this study. Thus the rates predicted by the linear model appear to match the observed microzooplankton growth rates more closely than those based on the published, non-linear (Q_10_) models. Further, the linear model corresponds to the idea that herbivorous protists respond to temperature similarly to autotrophs. However, our data cannot be used to support or reject either of the above approaches because the growth rates measured at >5°C in this study were apparently constrained by factors other than temperature.

### The Effect of Biotic Factors

As noted by Caron and Rose [71], the temperature-growth relationship plays itself out in nature together with several other factors, which affect the growth of phototrophs and heterotrophs. Resource availability is central among these factors. The negative relationship between protist species-specific growth rates and their initial abundances in the present study suggests that some of them may have reached their carrying capacity. Similar abundance-growth relationships were found for *M. rubrum* [51] and bloom-forming phytoplankton [72]. Prey availability also superseded temperature effects on microzooplankton dynamics in several field studies in Arctic and boreal waters [69], [73], [74]. Further, phytoplankton prey composition had pronounced effects on the growth and feeding rates of cultured Antarctic ciliates [63]. Total Chl may be too crude a measure to describe the specific resource requirements of individual microzooplankton species. However, heterotrophic ciliate average µ in the Barents Sea differed between samples with Chl <2 and >2 µg L^−1^ (0.37±0.06 and 0.55±0.06 d^−1^, respectively, p<0.05).

The faster growth of larger cells in this study apparently contradicts the allometric scaling equations (Table 2), which predict a continuous decrease of mass-specific growth rate with increasing size. Such deviations are not unusual in field experiments [37], [75], [76] and could be due to different growth conditions for protists depending on their size. Incubation experiments with natural plankton often yield net growth estimates for microzooplankton due to intraguild predation within their communities (e.g., [76]–[78]). For example, two small-sized ciliates, *Balanion comatum* and *Lohmaniella oviformis*, grew more slowly than predicted across a temperature gradient (75% of µ_max_ vs. 190% average for all ciliates and 122% for dinoflagellates) and did not correlate with their own initial abundance in our study. This lack of relationship may indicate that they were kept below their carrying capacity by predators or competitors. In the Fram Strait, small ciliates occasionally grew in diluted samples, where their encounter rate with potential predators was reduced, but not in whole seawater samples [15].

Predation could have also restricted ciliate growth at warmer temperatures (i.e., in AtW>5) in this study. Large tintinnids, such as *Parafavella gigantea* and thecate dinoflagellates, which are known to prey on ciliates and dinoflagellates [33], [79]–[81], were abundant in the southwestern Barents Sea during this study. Specifically, dinoflagellates from the genus *Dinophysis* prey on *M. rubrum* in search of kleptoplasts acquired previously by this ciliate from its cryptophyte prey [82]–[84]. In the absence of large diatoms, heterotrophic dinoflagellates, such as *Protoperidinium* spp., could have switched to ciliate prey. Copepod nauplii and the larvacean *Oikopleura*, which occurred in some of the samples collected in the Atlantic waters, can feed on and compete for food with ciliates [85], [86]. The lack of statistical difference between microzooplankton growth rates in the presence and absence of micro-metazoans (p = 0.6) does not exclude the possibility that metazoan grazing may have affected ciliates directly or indirectly in some of our experiments.

Caution should be exercised when applying the maximum species-specific growth rates observed in this study at the community level. On average, actively growing populations comprised 37% of total microzooplankton (43±9.8% and 35±10% in ArW and AtW, respectively). The rest of microzooplankton either declined or, in many cases, did not change significantly during the 24 h incubations. It is not surprising that only part of their community increased in most experiments in this study. Due to their competitive and/or predator-prey interactions and different resource requirements, multiple populations comprising microzooplankton may oscillate out of phase, whereas short-term incubations provide only a snapshot of these dynamics. The importance of a species-specific approach cannot be overemphasized in field growth rate experiments. Counting microzooplankton into size classes or broad taxonomic categories can mask dynamic processes within their communities and often yields net community growth rates of ∼0 in bottle experiments (i.e., growth and loss terms appear nearly balanced). Such equilibrium is unlikely to persist for any extended period of time in natural Arctic communities, where copepods can consume a large fraction of microzooplankton standing stocks daily (e.g., [74], [87]).

## Conclusions

The results of this study support the idea that microzooplankton play a major role in carbon cycling in the Arctic. These protists appear to be capable of growing as fast as their phytoplankton prey at extreme polar temperatures and demonstrate a remarkable ecological plasticity and resilience to environmental perturbations. Our data suggest that dynamic processes regulating plankton structure and function in the Arctic may be more complex than currently understood and will require additional field and laboratory research.
